# Collaborative Filtering for Brain-Computer Interaction Using Transfer Learning and Active Class Selection

**DOI:** 10.1371/journal.pone.0056624

**Published:** 2013-02-21

**Authors:** Dongrui Wu, Brent J. Lance, Thomas D. Parsons

**Affiliations:** 1 Machine Learning Laboratory, GE Global Research, Niskayuna, New York, United States of America; 2 Army Research Laboratory, Aberdeen Proving Ground, Aberdeen, Maryland, United States of America; 3 Department of Psychology, University of North Texas, Denton, Texas, United States of America; University of Adelaide, Australia

## Abstract

Brain-computer interaction (BCI) and physiological computing are terms that refer to using processed neural or physiological signals to influence human interaction with computers, environment, and each other. A major challenge in developing these systems arises from the large individual differences typically seen in the neural/physiological responses. As a result, many researchers use individually-trained recognition algorithms to process this data. In order to minimize time, cost, and barriers to use, there is a need to minimize the amount of individual training data required, or equivalently, to increase the recognition accuracy without increasing the number of user-specific training samples. One promising method for achieving this is collaborative filtering, which combines training data from the individual subject with additional training data from other, similar subjects. This paper describes a successful application of a collaborative filtering approach intended for a BCI system. This approach is based on transfer learning (TL), active class selection (ACS), and a mean squared difference user-similarity heuristic. The resulting BCI system uses neural and physiological signals for automatic task difficulty recognition. TL improves the learning performance by combining a small number of user-specific training samples with a large number of auxiliary training samples from other similar subjects. ACS optimally selects the classes to generate user-specific training samples. Experimental results on 18 subjects, using both 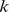 nearest neighbors and support vector machine classifiers, demonstrate that the proposed approach can significantly reduce the number of user-specific training data samples. This collaborative filtering approach will also be generalizable to handling individual differences in many other applications that involve human neural or physiological data, such as affective computing.

## Introduction

Future technologies that allow computer systems to adapt to individual users – or even to the current cognitive/affective state of the user – have many potential applications including entertainment, training, communication, and medicine. One promising avenue for developing these technologies is through brain-computer interaction (BCI) or physiological computing; i.e, using processed neural or physiological signals to influence human interaction with computers, environment, and each other [1], [2]. There are numerous challenges to effectively using these signals in system development. One of the primary challenges is the individual differences in neural or physiological response to tasks or stimuli. In order to address these individual differences, many researchers train or calibrate their systems for each individual, using data collected from that individual. However, the time spent collecting this data is likely to decrease the utility of these systems, slowing their rate of acceptance. As an example, one of the primary reasons that slow cortical potential-based BCIs never achieved mainstream acceptance, even among the disabled, is because using the slow cortical potential-based BCI could require training for several hour-long sessions per week for months in order to achieve satisfactory user performance [3].

While it is possible to train a generic model with group or normative data, in practice this tends to result in significantly lower performance than calibrating with individual data [4]. An example of this may be found in our earlier work [5], in which we used a support vector machine (SVM) to classify three task difficulty levels from neural and physiological signals while a user was immersed in a virtual reality based Stroop task [6], which has been shown to have high individual differences in neural and physiological response as the task difficulty varies [7]. Results revealed that when each subject is considered separately, an average classification rate of 96.5% can be obtained by SVM; however, the average classification rate was much lower (36.9%, close to chance) when a subject’s perception of task difficulty level was predicted using only data from other subjects. In a more recent study [8] on whether generic model works for rapid event-related potential (ERP)-based BCI calibration, a generic model was derived from 10 participants’ data and tested on the 11th participant. Experiments showed that seven of the 11 participants were able to use the generic model during online training, but the remaining four could not.

Novel approaches to analyses of individual differences have significant potential in helping to address these individual differences in neural and physiological responses [9], [10]. In particular, we are interested in analytical methods that decrease the amount of data required for training/calibration a customized BCI system, or equivalently, methods that increase the performance of the BCI system without increasing the number of user-specific training samples. Collaborative filtering – the process of making inferences about a user based on the combination of data collected from that user with a database of information collected from similar previous users – is one potential solution [11]. Using collaborative filtering to decrease the amount of time and data required for individual customization should, in turn, increase the usability leading to wider acceptance of BCI technologies [12].

This paper describes a successful collaborative filtering approach developed for implementation in a BCI system. While there are many types of BCI systems [1], the example application domain used herein was developed as a passive BCI (i.e. a BCI that uses a pattern recognition algorithm to passively monitor a user’s cognitive and/or affective state [13]), that would monitor a user of a virtual environment (VE) for cognitive assessment and rehabilitation, looking for neural and physiological indicators of task difficulty. The specific VE used for the sample domain is the Virtual Reality Stroop Task (VRST), which uses neuropsychological tests embedded into military-relevant VEs to evaluate potential cognitive deficits [6], [14], [15]. Cognitive assessment and rehabilitative VEs or serious games such as VRST require immersion on the part of the user to be successful [16]–[18]. One of the key aspects for immersion is the difficulty of the task being performed. If the task is too difficult, the user will become frustrated and lose interest. However, if the task is too easy, the user will become bored, again resulting in a loss of interest [19]–[21].

There are many ways to modulate difficulty based solely on the behavioral measures of user performance [22]. However, there are also strong individual differences in ability to handle difficulty, i.e., some users are better able to handle more difficult tasks than others. One way to address these individual preferences would be to combine information obtained from neural and physiological measures with the behavioral measures to provide superior performance for difficulty modulation [23]. However, these methods for neural and physiological-based difficulty modulation are strongly affected by the differences between individual physiological responses [2]. Thus, for these approaches to be successful, we will require a robust method for addressing the widely varying individual differences in physiological response to task difficulty.

**Figure 1:**
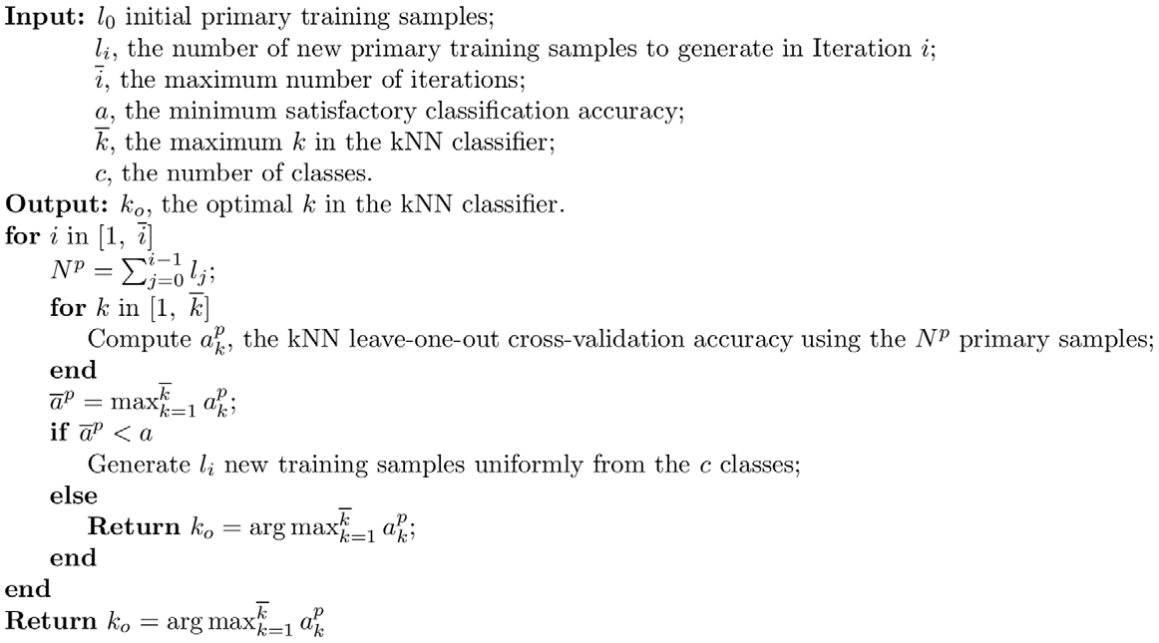
The baseline algorithm, in which only user-specific training samples are used, and new training samples are generated randomly from the 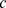
 **classes online.**

One method for tailoring a BCI pattern recognition algorithm for a specific user is to collect a set of user-specific training data samples at once, estimate the recognition performance using cross-validation, and iterate until the maximum number of iterations is reached, or the cross-validation performance is satisfactory. The pseudocode for such an algorithm is shown in Figure 1, where the 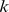-nearest neighbor (kNN) classifier is used for simplicity.

There are many techniques to improve this method. Two of them are examined in this paper:

- *Transfer Learning (TL)* [*24*] *: Use the information contained in other subjects’ training data.* Although training data from other subjects may not be completely consistent with a new subject’s profile, they may still contain useful information, as people may exhibit similar responses to the same task. As a result, improved performance can be obtained at recognizing the difficulty of a task.
- *Active Class Selection (ACS)* [*25*] *: Optimally generate the user-specific training samples online.* If in an application there are lots of offline unlabeled training samples and the bottleneck is to label them, then active learning [26]–[29] can be used to optimally select a small number of training samples to label. However, in many applications we do not have unlabeled data, and all training samples need to be generated online. Thus we cannot propose which samples to label; instead we must obtain additional training samples. So, the ACS problem becomes how to drive the selection of the class from which training samples are obtained during an online calibration session with the user, so that a high-performance classifier can be constructed from a small number of training samples.

In our previous research we have shown that TL can improve classification performance compared with a baseline that uses only the user-specific training samples [30], and ACS can improve classification performance compared with a baseline that selects the classes uniformly [31]. Because TL considers how to make use of data from other subjects and ACS considers how to optimally generate user-specific training samples online, they are independent and complementary. This paper presents theory and experimental results on a collaborative filtering approach which combine TL and ACS for learning an optimal classifier from a minimum amount of user-specific training data samples.

There has been some work on combining TL and collaborative filtering [32]–[36], where TL was used to make use of auxiliary data to address the data sparsity problem in collaborative filtering. Most of this work was for recommender systems, particularly movie recommendation. Our work is different from these in that: 1) we use TL to handle the data insufficiency problem instead of the sparsity problem; 2) we combine TL and ACS, instead of using TL only; and, 3) we apply our algorithm to a BCI system.

There are also a small number of existing collaborative filtering systems for BCI [8], [37]–[40], which integrate information from other users to improve the performance for the current user. For example, Lu et al. [40] built two classification models for each user, one is an adaptive user-specific model from user-specific online training data only, and the other is a user-independent model from offline training data from other users. The two models performed classifications independently for a new input, and the one with higher confidence score was chosen. Jin et al. [8] built an online genetic classification model by directly combining online user-specific training data and offline training data from other users. They showed that the online generic model achieved better performance than a generic model which used offline data from other users only. It also achieved similar performance to a typical model which used user-specific data only, but the online generic model needed less user-specific data so it was trained more quickly. Our work is different from these approaches in two aspects. First, we propose a different way to make use of the offline data from other users. Second, we propose an optimized procedure to generate the user-specific training data online.

## Methods

### Transfer Learning (TL)

This section introduces the theory and an algorithm for TL. For simplicity we use the kNN classifier as an example since it has only one parameter (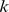) to optimize given a fixed distance function and the type of normalization. However, these ideas can be generalized to other classifiers such as the SVM [41].

#### TL theory

In many machine learning applications, in addition to the data for the current task, we also have data from similar but not exactly the same tasks. The learning performance can be greatly improved if these additional data are used properly. TL [24], [42] is a framework proposed for addressing this problem.

#### Definition (Transfer Learning)

[24] Given a source domain 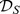 with learning task 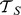, and a target domain 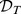 with learning task 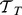, TL aims to help improve the learning of the target predictive function 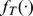 in 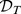 using the knowledge in 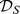 and 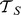, where 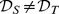, or 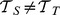.

In the above definition, a domain is a pair 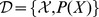, where 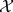 is a feature space and 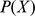 is a marginal probability distribution, in which 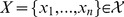. 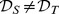 means that 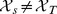, and/or 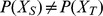, i.e., the features in the source domain and the target domain are different, and/or their marginal probability distributions are different. Similarly, a task is a pair 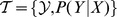, where 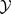 is a label space and 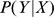 is a conditional probability distribution. 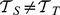 means that 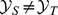, and/or 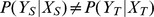, i.e., the label spaces between the source and target domains are different, and/or the conditional probability distributions between the source and target domains are different.

For example, in the domain of classifying the subjective difficulty level of a task in a VE based on neural and physiological signals, the labeled neural and physiological data from a user would be the primary data in the target domain, while the labeled neural and physiological data from other users would be the auxiliary data from the source domain. A single data sample would consist of the feature vector for a single epoch of neural and physiological data from one subject, collected as a response to a specific stimulus, and labeled with the difficulty of responding to that stimulus. Though the features in this primary data and auxiliary data would be the same, generally their marginal distributions are different, i.e., 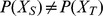, due to the fact that the baseline physiological levels for the subjects are likely to differ. Moreover, the conditional probabilities are also different, i.e., 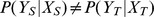, due to the significant individual differences in neural and physiological response to different difficulty levels. As a result, the auxiliary data from the source domain cannot represent the primary data in the target domain accurately, and must be integrated with some labeled primary data in the target domain to induce the target predictive function.

Previous work [42] has shown that when the primary training dataset is very small, training with auxiliary data can significantly improve classification accuracy, even when the auxiliary data is significantly different from the primary data. This result can be understood through a bias/variance analysis. When the size of primary training data is small, a learned classifier will have large variance and hence large error. Incorporating auxiliary data, which increases the number of training samples, can effectively reduce this variance. However, this data may increase the bias, since the auxiliary and primary training data have different distributions. This also suggests that as the amount of primary training data increases, the utility of auxiliary data should decrease [42].

#### TL algorithm

Suppose there are 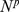 user-specific training samples 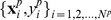 for the primary supervised learning problem, where 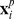 is the feature vector of the 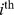 training sample and 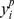 is its corresponding class label. The superscript 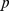 indicates the *primary* learning task. Additionally, there are 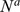 auxiliary training samples (training samples from other subjects) 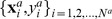, whose distribution is assumed to be similar to the primary training samples but not exactly the same. So, the auxiliary training samples should be treated as weaker evidence in designing a classifier. Moreover, we may want to select some “good” auxiliary training samples and discard the “bad” ones.

In the kNN classifier we need to optimize the number of NNs, 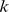. This is done through internal cross-validation [42], [43]. The most important parameter in determining the optimal 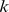 is the internal cross-validation accuracy on the primary training samples, i.e., the portion of the correctly classified primary training samples in the internal cross-validation, 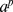. However, because 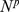 is very small, different 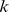 may easily result in the same 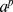. So, 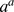, the internal cross-validation accuracy on the selected “good” auxiliary training samples, is used to break the ties. Once the optimal 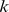 is identified for the kNN classifier, its performance can be evaluated as the accuracy of the algorithm classifying the test data.

**Figure 2:**
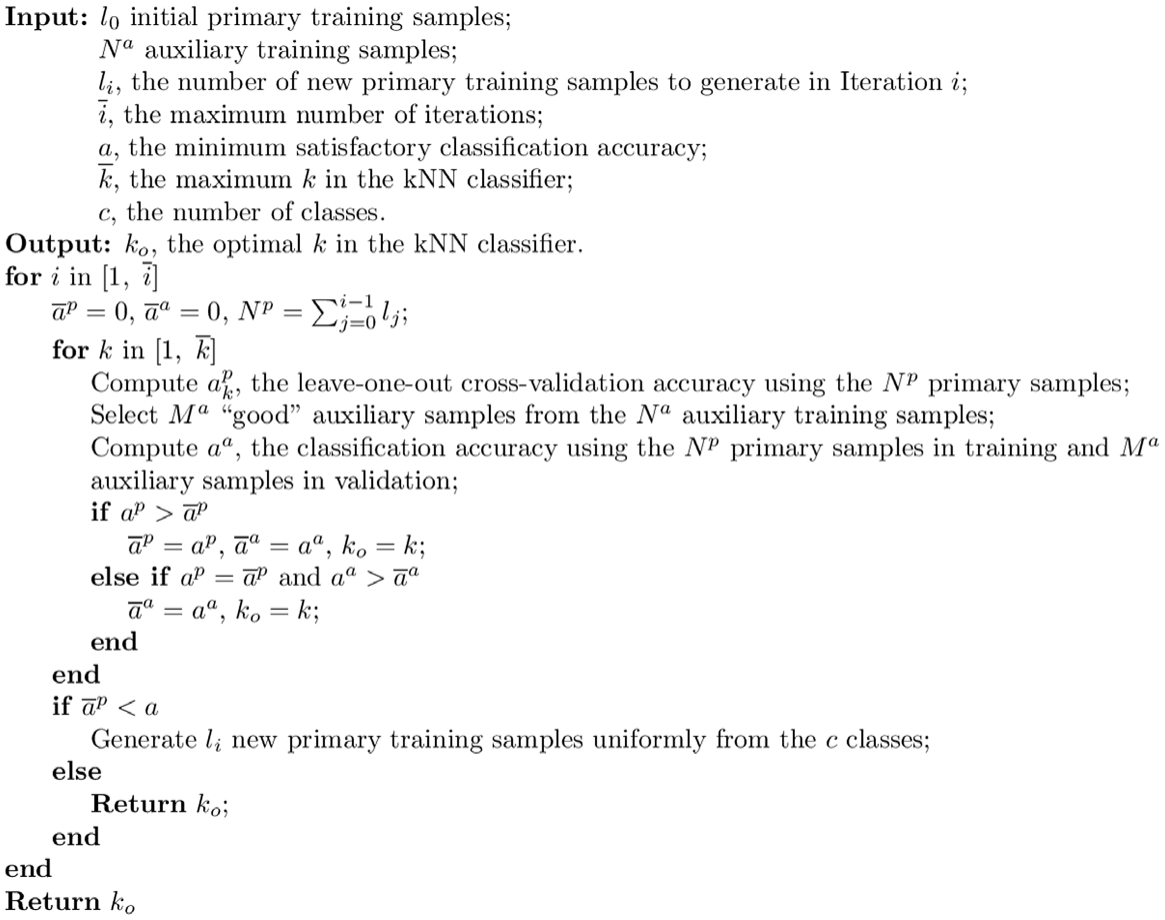
The TL algorithm, in which primary and auxiliary training samples are used together in determining the optimal 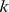
 **in the kNN classifier.**

As pointed out in [42], in many learning algorithms, the training data play two separate roles. One is to help define the objective function, and the other is to help define the hypothesis. Particularly, in kNN one role of the auxiliary data is to help define the objective function and the other is to serve as potential neighbors. In [30] we investigated both roles and found that using the auxiliary training samples in the validation part of the internal cross-validation algorithm generally achieved better performance. So, only this approach is considered in this paper. In each iteration the TL algorithm computes 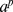 by leave-one-out cross-validation using the 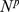 primary training samples, and 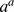 using the 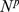 primary training samples to classify a selected set of “good” auxiliary training samples. Its pseudo-code is given in Figure 2, and is denoted TL in this paper.

How the “good” auxiliary training samples are selected is very important to the success of the TL algorithm. The general guideline is to select auxiliary training samples that are similar to the primary training samples. Specifically we are using a mean squared difference, calculated by:

1. Computing the mean feature vector of each class for the new subject, from the 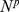 primary training samples. These are denoted as 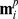, where 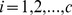 is the class index.
2. Computing the mean feature vector of each class for each subject in the auxiliary dataset. These are denoted as 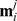, where 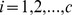 is the class index and 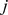 is the subject index.
3. Select the subject with the smallest difference from the new subject, i.e., 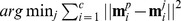, and use his/her data as auxiliary training data.

We note that the way to select “good” auxiliary training samples may be application dependent, and there are multiple potential avenues for research in this area. One of them is pointed out in the Future Research section.

### Active Class Selection (ACS)

This section introduces the theory and an algorithm for ACS. For simplicity the kNN classifier is used; however, the algorithm can be extended to other classifiers such as the SVM.

#### ACS theory

Active learning (AL) [26–26] has been attracting a great deal of research interest recently. It addresses the following problem: suppose that we have considerable amounts of offline unlabeled training samples and that the labels are very difficult, time-consuming, or expensive to obtain; which training samples should be selected for labeling so that the maximum learning (classification or prediction) performance can be obtained from the minimum labeling effort? For example, in speech emotion estimation [29], [44], [45], the utterances and their features can be easily obtained; however, it is difficult to evaluate the emotions they express. In this case, AL can be used to select the most informative utterances to label so that a good classifier or predictor can be trained based on them. Many different approaches have been proposed for AL [28] so far, e.g., uncertainty sampling [46], query-by-committee [47], [48], expected model change [49], expected error reduction [50], variance reduction [51], and density-weighted methods [52].

However, in many online applications we do not have large amounts of unlabeled offline data, and hence cannot propose which samples to label. Instead, it is possible to request more training samples on-the-fly from desired classes. For example, in the domain of classifying the subjective difficulty level of a task in a VE based on neural and physiological signals, there is no existing unlabeled data (i.e., neural/physiological signals), and it is not possible to generate sample training data with specific characteristics, such as a data sample with a specific heart rate, or EEG alpha power. However, we can control the difficulty level of the training sample. So, the problem of ACS is to optimally select the classes during real-time interaction with the user, by displaying stimuli from desired classes to the user in order to obtain training samples that allow a high-performance classifier to be constructed from minimal training samples.

Unlike the rich literature on AL, there has been limited research on ACS. Weiss and Provost [53] proposed a budget-sensitive progressive sampling algorithm for selecting training data. They considered a two-class classification problem. The proportion of Class 1 samples and Class 2 samples added in each iteration of the algorithm is determined empirically by forming several class distributions from the currently available training data, evaluating the classification performance of the resulting classifiers, and then determining the class distribution that performs best. They demonstrated that this heuristic algorithm performs well in practice, though the class distribution of the final training set is not guaranteed to be the best class distribution. Lomasky et al. [25] claimed that if one can control the classes from which training samples are generated, then utilizing feedback during learning to guide the generation of new training data may yield better performance than learning from any *a priori* fixed class distributions. They proposed several ACS approaches to iteratively select classes for new training instances based on the existing performance of the classifier, and showed that ACS may result in better classification accuracy. The below algorithm is based on and improves Lomasky et al.’s *Inverse* ACS algorithm [25].

#### ACS algorithm

In [31] we compared two ACS algorithms (*Inverse* and *Accuracy Improvement*), proposed by Lomasky et al. [25], with a baseline uniform sampling approach, and found that the *Inverse* algorithm consistently outperformed a baseline kNN classifier. This approach is considered and improved in this paper. The ACS method relies on the assumption that poor class accuracy is due to not having observed enough training samples. It requires internal cross-validation to evaluate the performance of the current classifier so that the class with poor performance can be identified and more training samples can be generated for that class.

We assume that there are 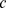 classes and no limits on generating instances of a particular class. The ACS algorithm begins with a small set of 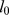 labeled training samples, where 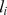 is the number of instances to generate in Iteration 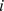. ACS is used to determine 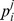 (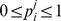), the portion of the 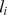 instances that should be generated from Class 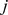. In Iteration 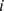, we record the classification accuracy (in the leave-one-out cross-validation) for each class, 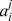, 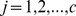. Then, Lomasky et al. defined the probability of generating a new instance from Class 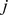 as:
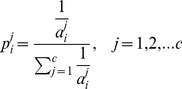
(1)i.e., it is proportional to the inverse of 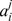. We have improved this approach by adding a constraint that no two consecutive new training samples can be generated from the same class, i.e., if the last new training sample is generated from Class 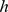, then the next new training sample is generated from Class 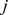 (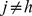) with probability:



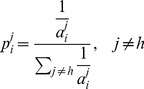
(2)This improvement reduces the risk that most new samples are generated from the class which has the lowest accuracy but is difficult to improve, and our experiments showed that it is more robust than Lomasky et al.’s original approach.

**Figure 3:**
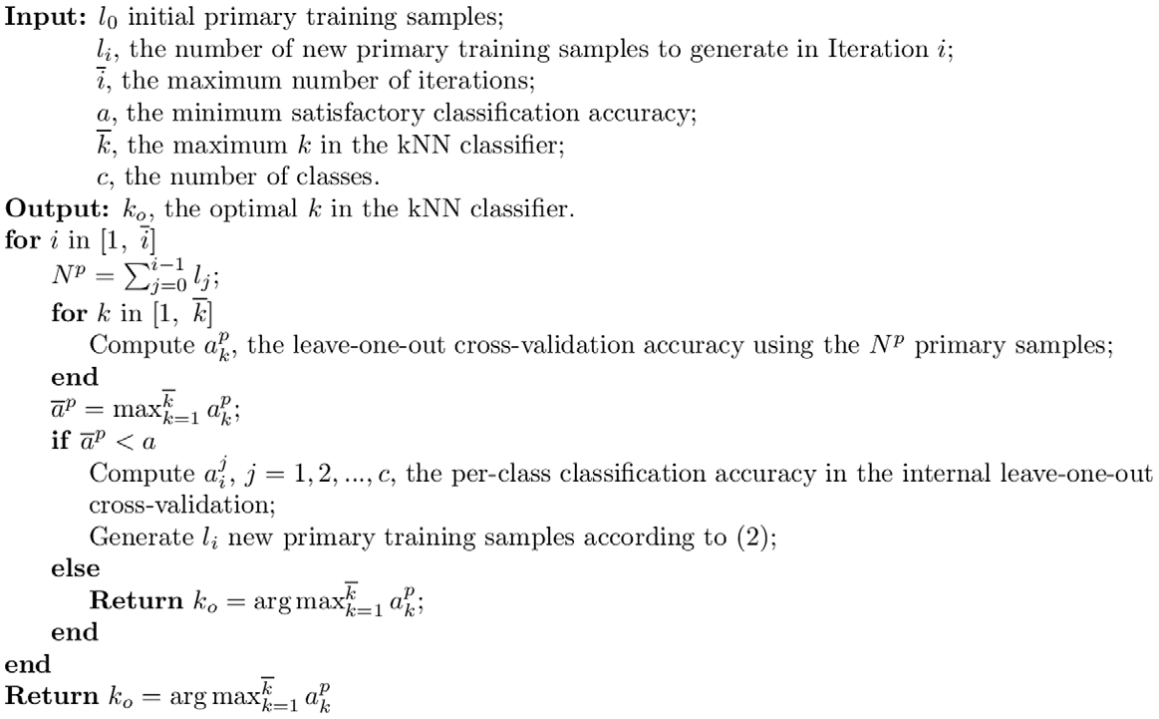
The ACS algorithm, in which the classes from which new training samples are generated are determined based on per-class cross-validation performance.

The detailed algorithm is given in Figure 3, and it is denoted ACS in this paper.

### Combining TL and ACS

**Figure 4:**
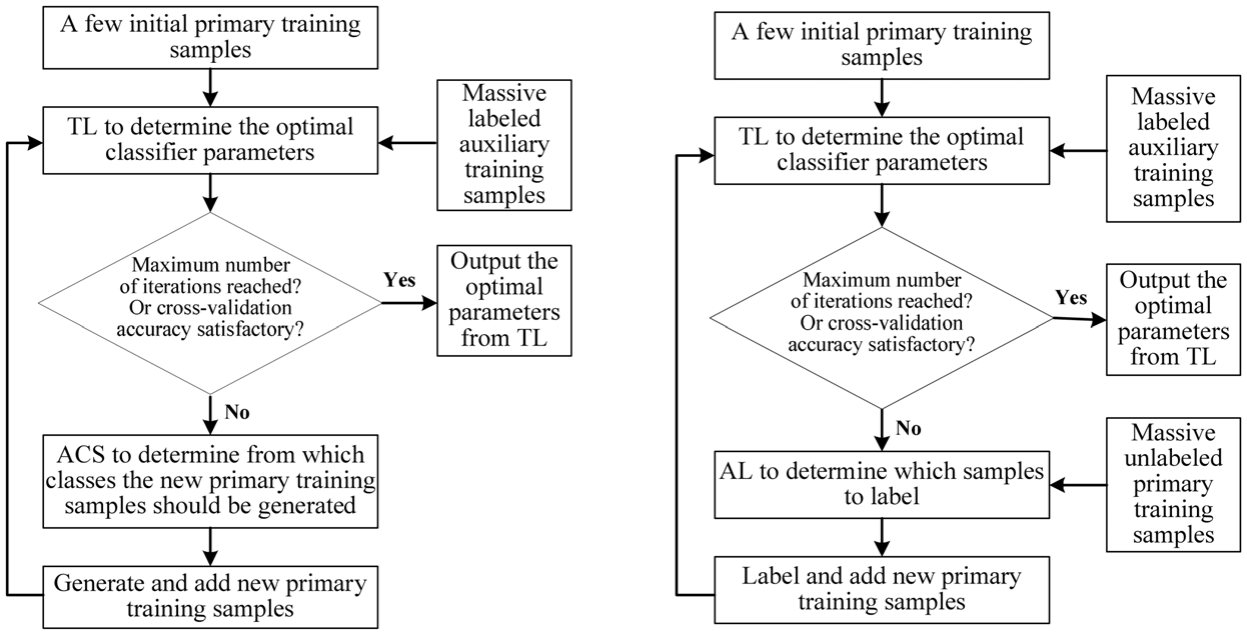
Methods to combine TL with ACS and AL. Left: Combining TL and ACS; Right: Combining TL and AL.

**Figure 5:**
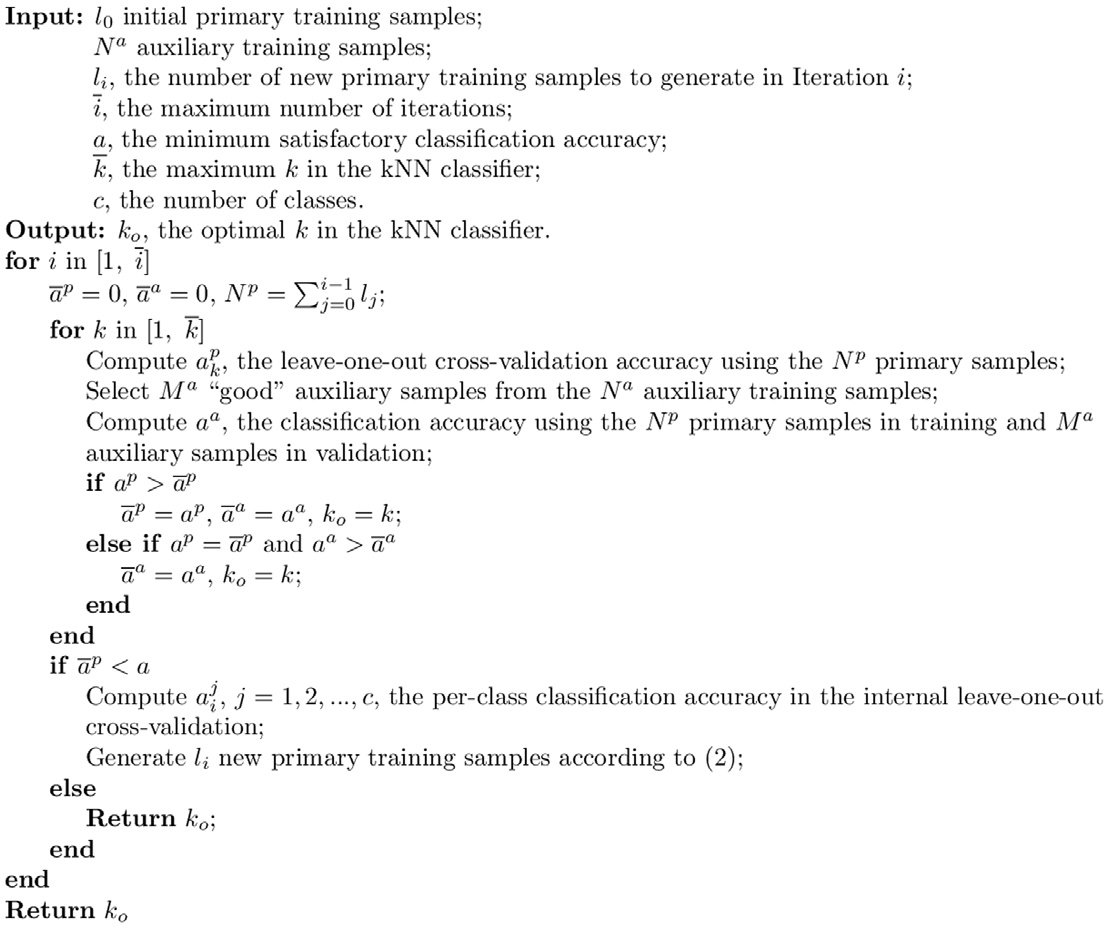
The TL+ACS algorithm, which uses TL to determine the optimal 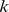
 **and ACS to generate new training samples.**

Because in our case TL considers how to make use of training data from other subjects and ACS considers how to optimally generate samples of user-specific training data online, they are independent and complementary. So, we conjecture that a collaborative filtering approach based on combining TL and ACS will result in improved classification performance. The fundamental concept is to use TL to select the optimal classifier parameters for the current subject based on available data obtained from the current subjects and other subjects, and then use ACS to obtain the most informative new training samples from the current subject, until the desired cross-validation accuracy is obtained, or the maximum number of training samples is reached, as illustrated in the left column of Figure 4. The pseudo-code for combining TL and ACS for a kNN classifier is given in Figure 5, and it is denoted TL+ACS in this paper.

The idea of TL+ACS can be illustrated with the following example. Suppose there are three label classes, and we start from 3 primary training samples (one for each class) and generate one new training sample in each iteration until the desired cross-validation accuracy is reached. In the first iteration, we use TL (combining the 3 primary training samples with a large number of “good” auxiliary training samples) to identify the optimal 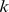, and then use ACS to compute the probability that the new training sample should be generated from each class. A new training sample is then generated according to the three probabilities. It is added to the primary training dataset. These 4 primary training samples are then combined with a large number of “good” auxiliary training samples and used in the second iteration. The program iterates until the desired cross-validation accuracy is reached. The optimal 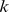 obtained in the last iteration (identified by TL) is output as the optimal kNN parameter.

Recall that ACS considers the case that we do not have unlabeled offline data in the target domain, and we can only control from which classes new training samples are generated on-the-fly. When there are large amounts of unlabeled data in the target domain and we want to suggest which ones to label, an AL approach is appropriate. As TL and AL are also independent and complementary, they could be combined in a similar fashion as TL and ACS [54], as shown in the right column of Figure 4.

## Experiments

This section presents our experimental results from a comparison of the four algorithms (baseline, TL, ACS, and TL and ACS combined, for both the kNN and SVM classifiers). These algorithms were applied to the task difficulty level classification problem introduced in [5]. We first consider kNN because we have shown that the specific TL and ACS approaches presented in the previous sections work well with this classifier [30], [31]. For example, in [30] we compared two TL approaches for the kNN classifier and found that the approach presented in this paper gave better results; in [31] we compared two ACS approaches for the kNN classifier and found that the approach presented in this paper gave better results. However, the generic framework of combining TL and ACS should apply to all classifiers, though the implementation details may differ. To demonstrate this, we also present results of these methods applied to an SVM classifier.

It is important to note that the purpose of the experiments is not to show how good a kNN or SVM classifier can be in task difficulty classification; instead, the goal was to demonstrate how TL and ACS, and especially their combination, can improve the performance of an existing classifier. The ideas proposed in this paper can also be extended to other classifiers, and also to other applications, including additional BCI, physiological computing, or affective computing systems [44].

### Experiment Setup and Data Acquisition

The data included in this paper were drawn from a larger study on the VRST [6]. Neural and physiological measures were used to predict levels of threat and task difficulty. The VRST is part of a battery of tests developed by Parsons that are found in an adaptive VE, which consists of a virtual city, a virtual vehicle checkpoint, and a virtual Humvee driving scenario in simulated Iraq and Afghanistan settings [14], [21], [55]–[57].

#### Ethics statement

The University of Southern California’s Institutional Review Board approved the study. Upon agreement to participate, prospective subjects were educated as to the procedure of the study, possible risks and benefits, and alternative options (non-participation). Prior to actual participation, they completed written informed consents approved by the University of Southern California’s Institutional Review Board. After each subjects’ written informed consent was obtained, basic demographic information was recorded.

#### Participants and procedure

A total of 20 college-aged subjects participated in the study. Two of the 20 subjects did not respond at all in one of the three scenarios, and were excluded as outliers. While experiencing the VRST, participant neural and physiological responses were recorded using a Biopac MP 150 system in conjunction with the NeuroSim Interface (NSI) software developed at Parsons’ Neuroscience and Simulation Laboratory (NeuroSim) at the University of Southern California. Electroencephalography (EEG), Electrocardiographic activity (ECG), Electrooculography (EOG), Electrodermal activity (EDA), and Respiration (RSP) were recorded. Following completion of the VRST protocol, none of the subjects reported simulator sickness.

EEG was measured using seven electrodes placed at locations Fp1, Fp2, Fz, Cz, Pz, O1, and O2 according to the international 10–20 system for EEG electrode placement. The EEG signal was recorded at 512 Hz, and was referenced to linked ear electrodes. EDA was measured using Ag/AgCl electrodes placed on the index and middle fingers of the non-dominant hand [58]. ECG was recorded with use of a Lead 1 electrode placement, with one Ag/AgCl electrode placed on the right inner forearm below the elbow, another in the same position on the left inner forearm, and a third on the left inner wrist to serve as a ground. Finally, RSP was recorded with a transducer belt placed around widest area of the rib cage.

#### Virtual Reality Stroop Task (VRST)

**Figure 6:**
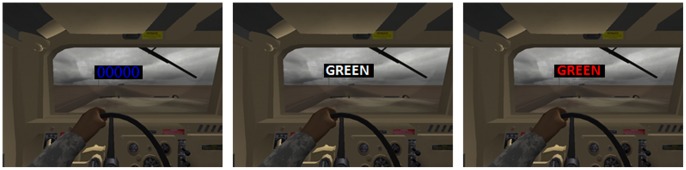
The Humvee Stroop scenarios. Left: Color naming; Middle: Word reading; Right: Interference.

The VRST involves the subject being immersed in a VE consisting of a Humvee that travels down the center of a road in a desert environment with military relevant events while Stroop stimuli appear on the windshield (see Figure 6). The VRST is a measure of executive functioning and was designed to emulate the classic Stroop test [59]. Like the traditional Stroop, the VRST requires an individual to respond by selecting one of three colors, (i.e., red, green, or blue). Unlike the traditional Stroop, a subject responds by pressing a computer key, and the VRST also adds a simulation environment with military relevant events in high and low threat settings. Participants interacted with the VRST through an eMagin Z800 head-mounted display (HMD). To increase the potential for sensory immersion, a tactile transducer was built by mounting six Aura bass shaker speakers on a three foot square platform.

#### Stimuli and design

Participants were immersed in the VRST as neural and physiological responses were recorded. EEG, ECG, EDA, and RSP were collected as participants rode in a simulated Humvee through alternating zones of low threat (i.e., little activity aside from driving down a desert road) and high threat (i.e., gunfire, explosions, and shouting amongst other stressors). The VRST was employed to manipulate levels of task difficulty. The VRST consisted of 3 conditions: 1) word-reading, 2) color-naming, and 3) Stroop interference. The Stroop interference condition displays a color word, such as red, in a different color font, while the subject’s task is to name the color of the font, not read the word (right column of Figure 6). Each Stroop condition was experienced once in a high threat zone and once in a low threat zone.

There are many different task difficulty levels in VRST. In this study we chose the following three:

- Scenario I: Low threat, color naming.
- Scenario II: High threat, color naming.
- Scenario III: High threat, Stroop interference.

Each scenario consisted of 50 stimuli. Three colors (Blue, Green, and Red) were used, and they were displayed randomly with equal probability. In Scenario I, 50 colored numbers were displayed one by one while the subject was driving through a safe zone. Scenario II was similar to Scenario I, except that the subject was driving through an ambush zone. Scenario III was similar to Scenario II, except that Stroop stimuli instead of color naming stimuli were used. In terms of task difficulty, the three scenarios are in the order of I 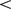 II 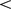 III. We set forth to classify these three scenarios using the proposed algorithms.

For each scenario each of the 50 stimuli was displayed at a random location on the windshield in a different color, randomly selected from one of the three different color schemes, in order to reduce signal habituation. Stimuli were presented for a maximum of 1.25 seconds each, and participants were asked to respond as quickly as possible without making mistakes. As shown in Table 1, the average reaction time was less than one second. The next stimulus was displayed when the user gave response to the current one. So, in total each stimulus took only a few seconds, and the 150 stimuli were finished in about 10 minutes.

**Table 1:** Mean and standard deviation of two performance measures in different scenarios.

	Mean			Standard Deviation		
Scenario	I	II	III	I	II	III
Number of correct responses	40.222	41.944	33.667	10.268	11.526	13.960
Reaction time (second)	0.805	0.776	0.866	0.072	0.076	0.089

### Comparison of the Algorithms

Each of the 18 subjects had 150 responses (50 stimuli for each task difficulty level). The same 29 features from our previous analysis [5] were used (shown in Table 2), with all 29 features being used across all subjects for this analysis. Twenty-one features were extracted from EEG, three from EDA, three from RSP, and two from ECG. Feature extraction consisted of segmenting the data into 3-second epochs that were time locked from 1 second prior to the stimulus occurrence to 2 seconds after. EOG artifacts were removed from the EEG using a standard regression-based approach. Then, EEG data was filtered using a [1], [30] Hz bandpass filter, epoched into overlapping 1 second windows, and detrended. Spectral power was then calculated in the theta [3.5, 7] Hz, alpha [7.5, 13.5] Hz, and beta [13.5, 19.5] Hz frequency bands for each channel. The EDA features were the mean, minimum, and maximum amplitude response in the epoch window. Respiration was scored similarly, with mean, minimum, and maximum amplitude in the epoch window. ECG features consisted of the number of heartbeats and the average inter-beat intervals (IBIs, scored as the time difference in seconds between successive R waves) in the epoched window. We normalized each feature for each individual subject to [0, 1].

**Table 2:** The 29 features used by the kNN and SVM classifiers.

								EEG																				
SCL			RSP			ECG		FP1			FP2			Fz			Cz			Pz			O1			O2		
min	max	mean	min	max	mean	Heartbeat	IBI	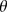	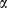	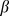	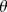	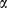	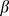	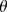	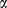	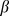	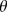	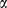	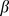	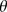	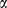	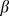	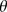	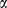	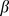	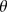	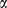	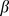

**Figure 7:**
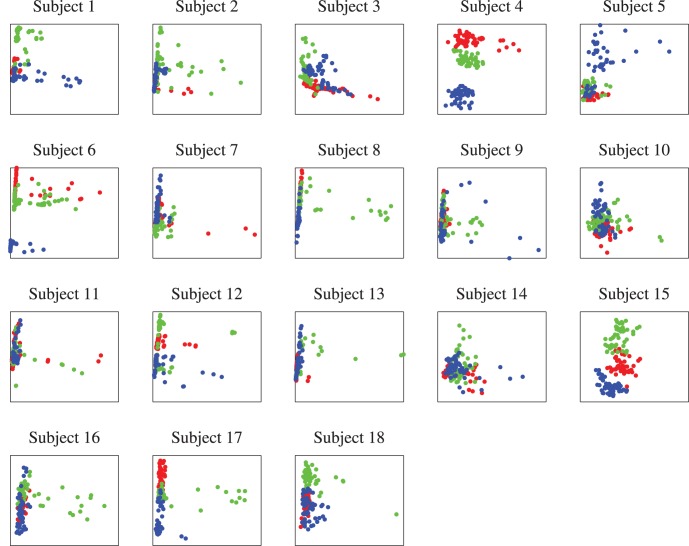
Coefficients of the first two principle components of the 29 features.

The coefficients of the first two principle components of the 29 features for the 18 subjects are shown in Figure 7. Different colors are used to denote different scenarios: red for Scenario I, green for Scenario II, and blue for Scenario III. Observe that generally the distributions of these coefficients are quite different among the subjects, which suggests that it may be impossible to find a generic classifier that works well for all subjects. This has been confirmed by our previous studies. In [5] we have reported that when we trained a SVM classifier on 17 subjects and tested it on the remaining subject, the average classification rate was 36.9%, close to chance. We also trained a kNN classifier on 17 subjects and tested it on the remaining subject. The average classification rate was 35.8%, again close to chance.

However, when examining Figure 7 more closely, we can observe that some subjects share similar distributions of the principle component coefficients, e.g., Subjects 4 and 6, Subjects 8 and 17, and Subjects 13 and 16. This suggests that auxiliary data from other subjects may be helpful in building a classifier for a new user. Next we show how classification performance can be improved above baseline using TL, ACS, and their combination.

#### kNN classification

In kNN classification we set the maximum number of primary training samples to 30, and 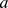, the minimum satisfactory classification accuracy, to 1, i.e., the algorithms terminated when 30 primary training samples were generated. The Euclidean distance was used to specify nearest neighbors. We studied each subject separately, and for each subject 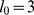 (so that there is at least one primary training sample for each labeled class). We used 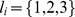 for 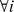, i.e., in the first experiment, only one primary training sample was generated in each iteration; in the second experiment, two primary training samples were generated in each iteration; and in the third experiment, three primary training samples were generated in each iteration. After Iteration 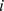, the kNN classification performance was evaluated using the remaining 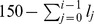 responses from the same subject. We repeated the experiment 100 times (each time selecting the 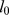 initial training samples randomly) for each combination of subject and 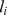, and then recorded the average performances of the four algorithms. It was necessary to repeat the experiment many times to ensure the statistical significant of the results. This is because there were two forms of randomness: 1) training data samples were selected randomly, so for the same sequence of class labels the training samples were different; and, 2) the class to select training samples from was chosen according to a probability distribution instead of deterministically.

**Figure 8:**
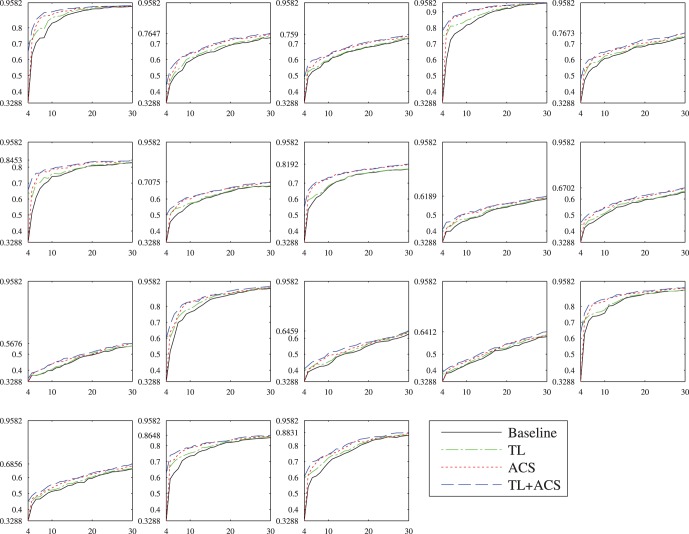
Performances of the four kNN classifiers on the 18 subjects for 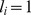
. The horizontal axis shows 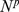, and the vertical axis shows the testing accuracy on the 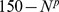 examples from the same subject.

Figure 8 shows the performances of the four algorithms on the 18 subjects for 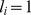. Observe that significantly different classification accuracies were obtained for different subjects. For example, with 30 user-specific training samples, 95.82% accuracy was obtained for Subject 4 by TL+ACS, but only 56.76% for Subject 11. However, regardless of the large individual differences, both TL and ACS outperformed the baseline for all 18 subjects, and TL+ACS achieved the best performance among the four.

**Figure 9:**
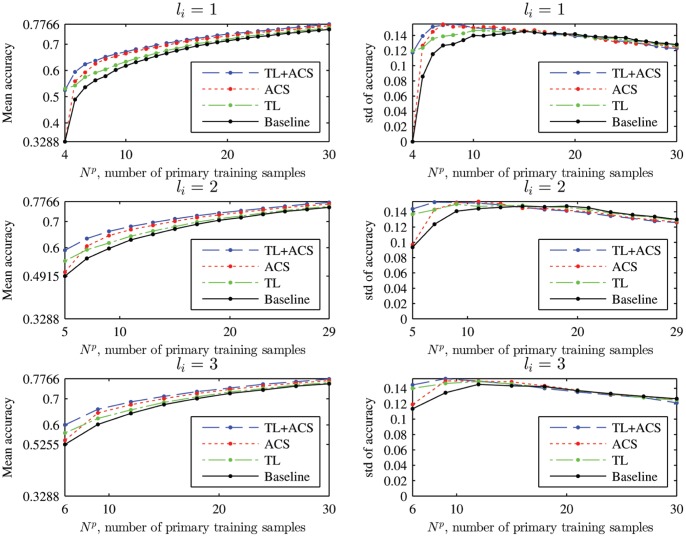
Mean and standard deviation (std) of the four kNN classifiers on the 18 subjects. 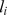
 is the number of primary training samples generated in each iteration.

The mean and standard deviation of the classification accuracy of the four algorithms for 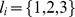 on the 18 subjects are shown in Figure 9. Observe that:

1. TL outperformed the baseline approach. The performance improvement is generally larger when 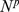 is small. As 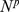 increases, the performances of TL and the baseline converge, i.e. the effect of auxiliary training data decreases as the number of primary training data samples increases.
2. ACS outperformed the baseline approach, and when 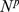 increased the performance improvement of ACS became larger than the performance improvement of TL over the baseline.
3. TL+ACS outperformed the other three approaches. It inherited both TL’s superior performance for small 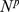 and ACS’s superior performance for large 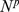, and showed improved performance overall.

To show that the performance differences among the four algorithms are statistically significant, we performed paired 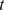-tests to compare their average accuracy (Table 3), using 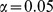. The results showed that the performance difference between any pair of algorithms is statistically significant (Table 4). Although our 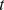-tests revealed significance, we decided to be very conservative with our results and took measures to ensure that the probability of Type I error did not exceed 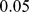. Hence, we also performed Holm-modified Bonferroni corrections [60] to assess classification accuracy through consideration of the four algorithms and three 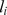 together. Despite the very conservative nature of Bonferroni correction, 10 of the 15 differences are statistically significant (the five insignificant ones are shown in bold in Table 4).

**Table 3:** Average classification accuracy for the four methods.

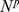																													
	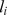	Method	4	5	6	7	8	9	10	11	12	13	14	15	16	17	18	19	20	21	22	23	24	25	26	27	28	29	30
		Baseline	.33	.49	.54	.56	.58	.60	.62	.63	.64	.65	.66	.68	.68	.69	.70	.71	.71	.72	.72	.73	.73	.74	.74	.75	.75	.75	.76
		TL	.53	.54	.57	.59	.60	.62	.63	.65	.65	.67	.68	.68	.69	.70	.71	.71	.72	.73	.73	.73	.74	.74	.75	.75	.75	.76	.76
	1	ACS	.33	.56	.59	.63	.64	.65	.66	.67	.68	.69	.70	.70	.71	.72	.72	.73	.73	.74	.74	.74	.75	.75	.76	.76	.76	.77	.77
		TL+ACS	.53	.59	.62	.64	.65	.66	.67	.68	.69	.70	.70	.71	.72	.72	.73	.73	.74	.74	.75	.75	.75	.76	.76	.77	.77	.77	.78
		Baseline		.49		.56		.60		.63		.65		.67		.69		.70		.71		.73		.74		.75		.75	
		TL		.55		.59		.62		.64		.66		.68		.70		.71		.72		.73		.74		.75		.76	
kNN	2	ACS		.51		.61		.65		.67		.69		.70		.71		.72		.73		.74		.75		.76		.77	
		TL+ACS		.59		.63		.66		.68		.70		.71		.72		.73		.74		.75		.76		.77		.77	
		Baseline			.53			.60			.64			.68			.70			.72			.73			.75			.76
		TL			.57			.62			.66			.69			.71			.73			.74			.75			.76
	3	ACS			.54			.65			.68			.70			.72			.74			.75			.76			.77
		TL+ACS			.60			.66			.69			.71			.73			.74			.76			.76			.77
		Baseline	.33	.47	.50	.53	.56	.58	.60	.62	.64	.65	.66	.67	.68	.69	.70	.71	.72	.72	.73	.74	.74	.75	.75	.76	.76	.77	.77
		TL	.53	.55	.57	.58	.60	.62	.63	.65	.66	.67	.68	.69	.70	.71	.72	.72	.73	.73	.74	.75	.75	.76	.76	.77	.77	.77	.78
	1	ACS	.33	.53	.55	.58	.60	.62	.63	.64	.65	.66	.67	.69	.69	.70	.71	.72	.72	.73	.74	.74	.75	.75	.76	.76	.76	.77	.77
		TL+ACS	.52	.58	.63	.64	.66	.67	.68	.69	.70	.71	.72	.72	.73	.74	.74	.75	.75	.76	.76	.77	.77	.78	.78	.78	.79	.79	.79
		Baseline		.47		.54		.59		.62		.65		.68		.70		.71		.73		.74		.75		.76		.77	
		TL		.55		.59		.62		.65		.67		.69		.71		.73		.74		.75		.76		.77		.77	
SVM	2	ACS		.49		.56		.61		.64		.66		.68		.70		.72		.73		.74		.75		.76		.77	
		TL+ACS		.58		.64		.67		.69		.71		.73		.74		.75		.76		.77		.78		.78		.79	
		Baseline			.50			.59			.64			.68			.70			.72			.74			.76			.77
		TL			.57			.62			.66			.69			.72			.74			.75			.77			.78
	3	ACS			.51			.61			.65			.68			.71			.73			.75			.76			.77
		TL+ACS			.60			.66			.70			.72			.74			.76			.77			.78			.79

**Table 4:** Paired 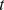-test results (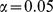) on classification accuracy.

		TL vs Baseline			ACS vs Baseline			TL+ACS vs Baseline			TL+ACS vs TL			TL+ACS vs ACS		
	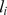	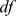	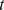	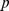	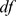	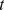	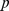	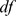	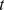	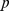	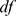	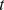	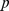	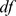	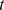	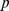
	1	26	2.69	**0.0123**	26	8.01	0.0000	26	6.09	0.0000	26	9.48	0.0000	26	2.21	**0.0364**
kNN	2	12	3.24	0.0071	12	7.34	0.0000	12	6.01	0.0001	12	9.11	0.0000	12	2.65	**0.0213**
	3	8	2.87	**0.0207**	8	5.84	0.0004	8	5.08	0.0010	8	7.64	0.0001	8	2.35	**0.0467**
	1	26	3.77	0.0008	26	4.54	0.0001	26	7.46	0.0000	26	10.00	0.0000	26	6.74	0.0000
SVM	2	12	3.89	0.0022	12	6.20	0.0016	12	6.20	0.0000	12	8.94	0.0000	12	6.58	0.0000
	3	8	3.04	0.0161	8	2.69	0.0273 5	8	5.18	0.0008	8	7.98	0.0000	8	5.87	0.0004

The 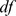 for 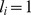 is 26 because there are 27 different 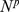 in this case 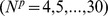. The 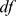 for 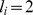 is 12 because there are 13 different 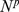 in this case 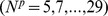. The 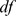 for 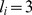 is 8 because there are 9 different 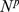 in this case 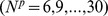.

**Figure 10:**
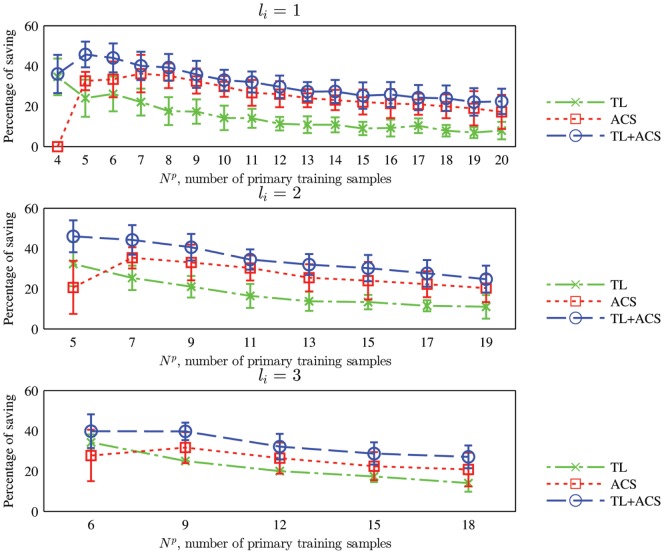
Mean and standard deviation of the percentage of primary training samples saved by TL, ACS, and TL+ACS over the baseline approach, when the kNN classifier is used. 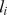
 is the number of primary training samples generated in each iteration.

We have shown that with the same number of primary training samples, TL, ACS, and TL+ACS can give higher classification accuracy compared with the baseline approach. As an equivalent goal of the improved algorithms is to learn an optimal classifier using a minimum number of primary training samples, it is also interesting to study how many primary training samples can be saved by using the three improved algorithms, as compared to the baseline approach. Take TL as an example. Assume that the TL algorithm has a classification accuracy of 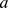 when 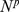 primary training samples are used. We then find how many primary training samples are needed by the baseline algorithm to achieve the same classification accuracy and denote that number by 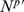. Then 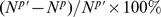 is percentage of primary training samples saved by TL. The mean and standard deviation of the percentages are shown in Figure 10. Note that we only show 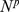 up to 20 because 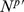 is larger than 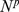 and the maximal 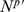 in the experiments is 30. Observe from Figure 10 that:

1. TL can save over 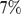 primary training samples over the baseline approach, especially when 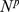 is small. When 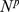 increases the saving becomes smaller, which is intuitive, as more primary training samples diminish the usefulness of the auxiliary training samples.
2. ACS can save over 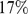 primary training samples over the baseline, especially when 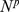 is small. When 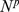 increases the saving generally becomes smaller, which is intuitive, as the classifier converges to the optimal one when each class has sufficient training samples, no matter how they are generated.
3. TL+ACS can save over 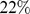 primary training samples over the baseline, especially when 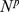 is small. It also outperformed both TL and ACS.

To show that the percentages of saved primary training samples among the four algorithms are statistically significant, we performed paired 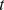-tests to compare their average savings (Table 5), using 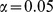. The results showed that the percentage of saved primary training samples between any pair of algorithms is statistically significant (Table 6). Again, to ensure that the probability of Type I error does not exceed 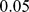, we also performed Holm-modified Bonferroni correction on the percentages by considering the four algorithms and three 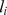 together. The results indicate that all 15 differences are statistically significant.

**Table 5:** Average percentage of saved primary training samples over the baseline method.

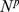																			
	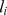	Method	4	5	6	7	8	9	10	11	12	13	14	15	16	17	18	19	20
		TL	34.60	24.01	26.10	22.14	17.60	17.35	14.25	14.04	11.36	10.84	10.80	8.97	9.25	10.39	7.87	6.99	7.95
	1	ACS	0	32.54	33.35	36.23	35.14	32.53	29.85	26.68	25.99	24.03	23.10	22.14	21.35	21.01	19.97	19.00	17.21
		TL+ACS	36.05	45.74	44.01	40.03	39.30	35.82	32.97	32.03	29.68	27.25	27.36	25.28	25.82	24.31	23.97	22.14	22.41
		TL		32.28		25.36		20.98		16.41		13.73		13.31		11.47		10.99	
kNN	2	ACS		20.63		35.34		33.05		30.25		25.41		23.96		22.21		20.27	
		TL+ACS		46.05		44.25		40.61		34.51		31.98		30.19		27.60		24.75	
		TL			34.26			25.00			20.00			17.33			14.09		
	3	ACS			27.78			31.67			26.46			22.45			20.81		
		TL+ACS			39.81			39.72			32.17			28.74			27.09		
		TL	45.95	37.86	32.71	27.79	22.02	20.11	16.82	17.30	17.33	15.27	16.81	14.07	12.86	12.75	11.61	10.17	11.23
	1	ACS	3.33	35.68	25.99	26.46	22.18	20.01	17.04	13.62	12.14	12.57	12.87	10.59	5.42	3.98	3.00	1.15	4.25
		TL+ACS	44.88	48.11	50.34	45.57	44.20	40.35	38.91	37.00	34.75	33.35	30.84	29.17	26.43	24.74	25.46	23.23	21.33
		TL		40.28		32.44		27.09		19.77		16.17		16.21		13.03		13.15	
SVM	2	ACS		24.69		20.54		16.94		17.44		13.38		8.08		6.68		5.11	
		TL+ACS		52.13		48.40		40.71		38.02		34.13		31.67		29.06		26.60	
		TL			37.96			27.50			23.46			17.37			15.55		
	3	ACS			31.48			23.06			12.22			8.80			6.63		
		TL+ACS			46.48			41.30			36.55			31.96			28.10		

**Table 6:** Paired 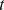-test results (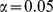) on percentage of saved primary training samples.

		TL vs Baseline			ACS vs Baseline			TL+ACS vs Baseline			TL+ACS vs TL			TL+ACS vs ACS		
	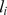	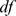	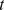	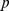	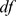	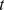	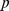	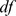	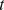	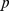	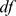	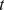	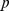	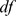	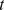	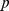
	1	16	7.98	0.0000	16	11.63	0.0000	16	17.06	0.0000	16	15.36	0.0000	16	3.43	0.0034
kNN	2	7	6.75	0.0003	7	12.91	0.0000	7	12.61	0.0000	7	21.45	0.0000	7	3.49	0.0101
	3	4	6.29	0.0033	4	13.35	0.0002	4	12.48	0.0002	4	7.31	0.0019	4	6.62	0.0027
	1	16	8.16	0.0000	16	5.65	0.0000	16	15.54	0.0000	16	11.07	0.0000	16	15.28	0.0000
SVM	2	7	6.30	0.0004	7	5.68	0.0008	7	11.69	0.0000	7	19.40	0.0000	7	23.49	0.0000
	3	4	6.07	0.0037	4	3.49	0.0250	4	11.29	0.0004	4	11.86	0.0003	4	12.00	0.0003

The 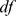 for 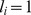 is 16 because there are 17 different 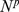 in this case 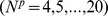. The 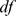 for 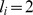 is 7 because there are 8 different 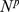 in this case 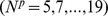. The 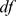 for 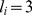 is 4 because there are 5 different 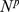 in this case 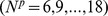.

#### SVM classification

To demonstrate that the generic framework of combining TL and ACS can be applied to other classifiers, we also implemented it for an SVM classifier [41], [61] using 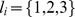. The same 29 features in the kNN classifier were again used in the experiment, and no feature selection was performed. The radial basis function (RBF) SVM in LIBSVM [62] was employed, so in training we tuned two parameters: 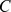, which is the penalty parameter of the error term, and 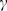, which defines the RBF kernel. Modification to the algorithms is very simple: In the TL part of Algorithms 2 and 4, instead of optimizing 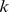 in kNN, we now optimize 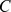 and 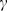.

**Figure 11:**
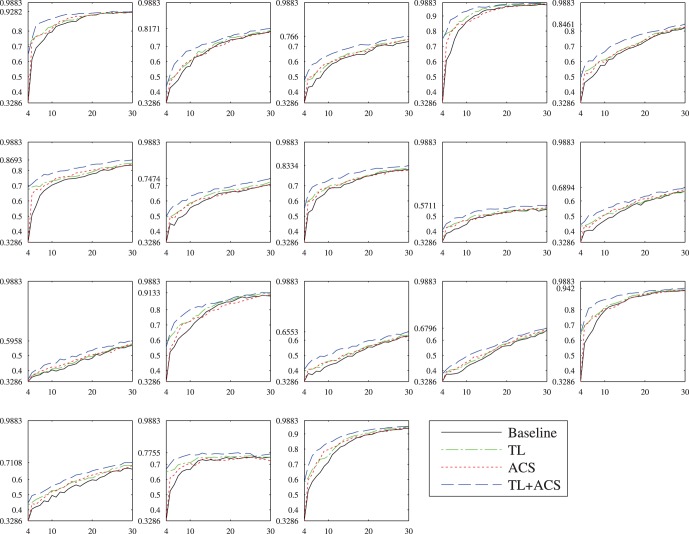
Performances of the four SVM classifiers on the 18 subjects for 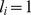
 **.** The horizontal axis shows 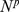, and the vertical axis shows the testing accuracy on the 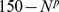 examples from the same subject.

Figure 11 shows the performances of the four algorithms on the 18 subjects for 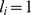. Again, significantly different classification accuracies were obtained for different subjects. However, regardless of the large individual differences, generally both TL and ACS outperformed the baseline for all 18 subjects, and TL+ACS achieved the best performance among the four.

**Figure 12:**
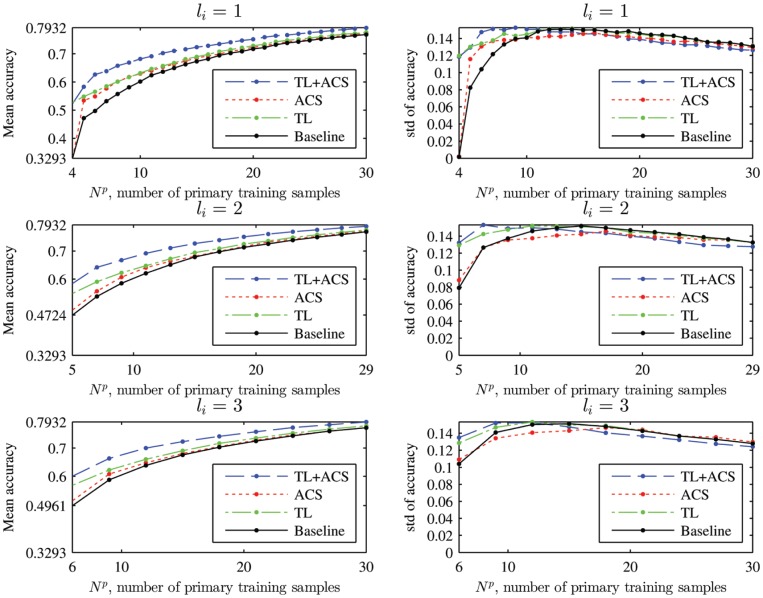
Mean and standard deviation (std) of the four SVM classifiers on the 18 subjects. 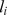
 is the number of primary training samples generated in each iteration.

The mean and standard deviation of the classification accuracy of the four algorithms for 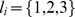 on the 18 subjects are shown in Figure 12. Observe that the average performances of TL, ACS and TL+ACS were all better than the baseline approach, which verified the effectiveness of the proposed approaches. However, unlike in Figure 9, where ACS outperformed TL when 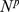 was large, for SVM classifier generally the performance of ACS was always worse than TL. This is because the ACS approach used in our experiment (selecting a new class according to the inverse of the per-class cross-validation accuracy, which is called *Inverse* in [25]), which is suitable for the kNN classifier, is not optimal for the SVM classifier. This fact is confirmed by Figure 1(b) in [25], where several ACS approaches for SVM were compared. In the future we will investigate better ACS approaches for the SVM classifier.

To show that the performance differences among the four algorithms are statistically significant, we performed paired 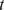-tests to compare their average accuracy (Table 3), using 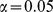. The results showed that the performance difference between any pair of algorithms is statistically significant (Table 4). To ensure that the probability of Type I error does not exceed 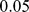, we also performed Holm-modified Bonferroni correction on the classification accuracy by considering the four algorithms and three 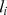 together. The results indicate that all 15 differences are statistically significant, despite the very conservative nature of Bonferroni correction.

**Figure 13:**
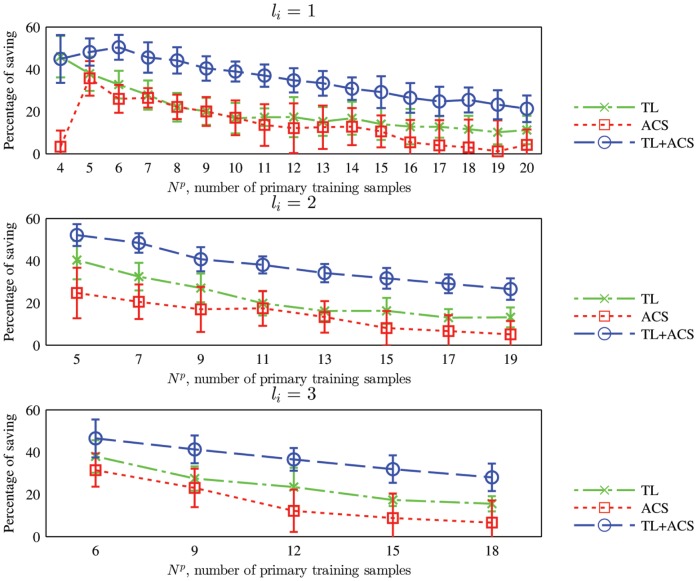
Mean and standard deviation of the percentage of primary training samples saved by TL, ACS, and TL+ACS over the baseline approach, when the SVM classifier is used. 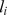
 is the number of primary training samples generated in each iteration.

Similar to the kNN case, for the SVM classifier we also studied how many percentages of primary training samples can be saved by using the three improved algorithms, compared with the baseline approach. The results are shown in Figure 13. TL+ACS can save over 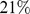 of primary training samples.

To show that the percentages of saved primary training samples among the four algorithms are statistically significant, we performed paired 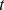-tests to compare their average savings (Table 5), using 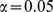. The results showed that the percentage of saved primary training samples between any pair of algorithms is statistically significant (Table 6). Again, to ensure that the probability of Type I error does not exceed 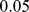, we performed Holm-modified Bonferroni correction on the percentages by considering the four algorithms and three 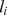 together. The results indicate that all 15 differences are statistically significant.

**Figure 14:**
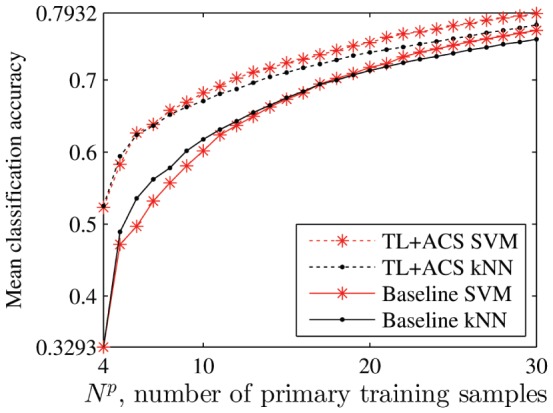
Comparison of the kNN and SVM classifiers for 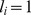
 **.**

As the search space of the SVM classifier is much larger than that of the kNN classifier, which implies that more primary training samples are needed to identify the optimal SVM model parameters, we expect that more significant performance improvement can be demonstrated for the SVM classifier by using the improved algorithms. The conjecture is clearly verified in Figure 14, in which the mean baseline and TL+ACS performances for 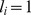 for both kNN and SVM are shown. We would also expect that the baseline SVM classifier should outperform the baseline kNN classifier as it is more sophisticated; however, Figure 14 shows that this is only true when 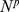 is large, because a small number of primary training samples are not sufficient to identify the optimal SVM parameters in a large search space. In summary, it seems that TL+ACS is particularly advantageous when a sophisticated classifier with a large search space is used.

## Discussion

We have demonstrated that a collaborative filtering approach based on TL and ACS can improve kNN and SVM classifier performance over a baseline classifier using the same quantity of training data, and that combining TL and ACS can achieve an even larger performance improvement. So, for same level of classification accuracy, TL+ACS may require a smaller number of user-specific training samples. This will reduce the data acquisition effort to customize an automatic task difficulty recognition BCI system, and hence increase its usability and popularity.

The VRST application is a scenario reflecting the ways in which this collaborative filtering algorithm could be used in automatic task difficulty classification. The VE maintains a database with many different subjects and their neural and physiological responses at different task difficulty levels. A new user can build his/her profile for automatic task difficulty classification by performing a calibration task, which is a subset of the primary task. Assuming that there are 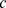 levels of task difficulty, the VE will display 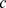 stimuli, one from each difficulty level. The neural and physiological responses from the user, along with selected “good” auxiliary training samples from the subject database, are then used by TL to identify the optimal parameters for the classifier. The TL module also computes the classification accuracy from cross-validation using these optimal parameters.

If the classification accuracy is not satisfactory, the ACS module then determines from which difficulty level the next stimulus should be displayed. The VE generates the corresponding stimulus and records the user’s neural and physiological responses during the test and adds it to the primary training sample database. The TL module is used again to update the optimal parameters for the classifier and compute the cross-validation accuracy. If the accuracy is satisfactory, then the VE configures the classifier using the optimal parameters and stops training; otherwise, it calls the ACS module to generate another new primary training sample from the user and iterates the process. The advantage is that the user will need to provide many fewer responses with TL+ACS than without, saving the user’s time calibrating the system.

Note that TL and ACS each requires a higher computational cost than the baseline approach, because TL needs to consider the auxiliary training samples in the internal cross-validation, and ACS needs to compute the per-class cross-validation accuracy. However, since the extra computational cost only occurs during the training process, it does not hinder the applicability of these improvements.

### Conclusions and Future Research

Individual differences make it difficult to develop a generic BCI algorithm whose model parameters fit all subjects. It is hence important to customize the BCI algorithm for each individual user by adapting its parameters using user-specific training data. However, collecting user-specific data is time-consuming and may also decrease the user’s interest in the BCI system. In this paper we have shown how TL, ACS, and a collaborative filtering approach based on their combination, can help learn an optimal classifier using a minimum amount of user-specific training data. TL exploits the information contained in auxiliary training data, and ACS optimally selects the class new training data to generate from. This approach reduces the data acquisition effort in customizing a BCI system, improving its usability and potentially, its popularity.

In the future we will improve both TL and ACS, thereby improving our collaborative filtering framework. For TL, we may be able to improve the selection of “good” auxiliary data by removing inconsistent data samples from the auxiliary data (i.e., reduce the intra-individual difference). If a subject cannot reliably classify his/her own perception of task difficulty, then unlikely his/her data can give good suggestions on another subject’s perception. One possible approach is that for each subject in the auxiliary data, we remove a minimum number of confusing data so that a 100% accurate classifier can be obtained. The remaining data from all subjects can then be combined to form the auxiliary dataset. To improve ACS, we will investigate other ACS approaches, such as *Redistricting* and *Improvement* [25].

Another direction of our future research is to integrate TL and ACS with feature selection. As it has been shown in [5], many of the 29 features are not useful. However, the useful features are subject dependent. As these features directly affect the classification performance and computational cost, it is necessary to integrate TL and ACS with feature selection for further performance improvement. In addition, we are involved in several large-scale (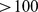 subjects) neural and physiological data collections, and intend to use that data to continue to refine and improve these collaborative filtering approaches.

Finally, we are interested in studying whether the proposed approach can also be used in cross-domain knowledge transfer [63], [64], e.g., whether the labeled task difficulty data in VRST can help improve the task difficulty recognition performance in other related application domains like personalized learning and affective gaming [65]–[67].
